# Sentinel Lymph Node in Aged Endometrial Cancer Patients “The SAGE Study”: A Multicenter Experience

**DOI:** 10.3389/fonc.2021.737096

**Published:** 2021-10-19

**Authors:** Stefano Cianci, Andrea Rosati, Virginia Vargiu, Vito Andrea Capozzi, Giulio Sozzi, Alessandro Gioè, Salvatore Gueli Alletti, Alfredo Ercoli, Francesco Cosentino, Roberto Berretta, Vito Chiantera, Giovanni Scambia, Francesco Fanfani

**Affiliations:** ^1^ Department of Gynecologic Oncology and Minimally-invasive Gynecologic Surgery, Università degli studi di Messina, Policlinico G. Martino, Messina, Italy; ^2^ Department of Woman and Child Health and Public Health, Division of Gynecologic Oncology, Fondazione Policlinico Universitario A. Gemelli, Istituto di Ricovero e Cura a Carattere Scientifico (IRCCS), Rome, Italy; ^3^ Department of Gynecologic Oncology, Gemelli Molise, Campobasso, Italy; ^4^ Department of Gynecology and Obstetrics, Università di Parma, Parma, Italy; ^5^ Department of Gynecologic Oncology, Aziende di Rilievo Nazionale di Alta Specializzazione (ARNAS) Civico Di Cristina Benfratelli, Palermo, Italy; ^6^ Department of Gynecologic Oncology, Università degli studi del Molise, Campobasso, Italy; ^7^ Department of Gynecologic Oncology, Università di Palermo, Palermo, Italy; ^8^ Department of Women and Child Health and Public Health, Università Cattolica del Sacro Cuore, Rome, Italy

**Keywords:** endometrial cancer, sentinel lymph node (SLN), aged population, elderly, lymphatic anatomy, indocyanine green

## Abstract

**Objective:**

The incidence of endometrial cancer is increasing in elderly people. Considering that aging progressively affects lymphatic draining function, we aimed to define its impact on IGC uptake during SLN mapping.

**Methods:**

A multicenter retrospective cohort of endometrial cancer patients with apparently early-stage endometrial cancer undergoing complete surgical staging with SLN dissection was identified in four referral cancer centers from May 2015 to March 2021. Patients were classified in Group 1 (<65 years old) and Group 2 (≥65 years old). The primary endpoint was the assessment of the overall, bilateral, and unsuccessful SLN mapping in the two groups. Secondary outcomes were the evaluation of SLN anatomical distribution and the identification of predictors for mapping failure applying a logistic regression.

**Results:**

A total of 844 patients were enrolled in the study (499 in Group 1 and 395 in Group 2). The overall detection rate, the successful bilateral mapping, and the mapping failure rate of the SLN were 93.8% *vs*. 87.6% (*p* = 0.002), 77.1% *vs*. 66.8% (*p* = 0.001), and 22.9% *vs*. 33.2% (*p* = 0.001), respectively, in Group 1 *vs*. Group 2. The advanced age affects the anatomical distribution of the SLN leading to a stepwise reduction of “unexpected” mapping sites (left hemipelvis: *p* < 0.001; right hemipelvis: *p* = 0.058). At multivariate analysis age ≥ 65 (OR: 1.495, 95% CI: 1.095–2.042, *p* = 0.011), BMI (OR: 1.023, 95% CI: 1.000–1.046, *p* = 0.047), non-endometrioid histotype (OR: 1.619, 95% CI: 1.067–2.458, *p* = 0.024), and LVSI (OR: 1.407, 95% CI: 1.010–1.961, *p* = 0.044) represent independent predictors of unsuccessful mapping. Applying binomial logistic regression analysis, there was a 1.280-fold increase in the risk of failed mapping for every 10-year-old increase in age (OR: 1.280, 95% CI: 1.108–1.479, *p* = 0.001). A higher rate of surgical under-staging (0.9% *vs*. 3.3%, *p* = 0.012) and adjuvant undertreatment (*p* = 0.018) was reported in Group 2.

**Conclusions:**

Old age represents a risk factor for SLN mapping failure both intrinsically and in relation to the greater incidence of other independent risk factors such as LVSI, non-endometrioid histotype, and BMI. Surgeons should target the usual uptake along UPP during the SLN dissection in this subgroup of patients to minimize mapping failure and the consequent risk of surgical under-staging and adjuvant undertreatment.

## Introduction

Endometrial carcinoma (EC) is a prevalent condition in the elderly with a mean age at diagnosis of 63 years old (1, 2).

The probability to develop an EC progressively increases with aging, starting from a risk of 0.77% at ages 40–59, and increasing to 0.87% at ages 60–69 or 1.24% at age >70 years (3).

Furthermore, 45.1% of women diagnosed with EC are ≥ 65 years old, with most of cancer-specific mortality clustered in this subgroup of patients (68.1%) (4).

The incidence of EC is bound to increase in elderly people; indeed, the European Union has reported the highest rate of people aged over 65 worldwide: to date, approximately 20% and forecast to boost up to 30% in 2060 (5).

Thus, in the future, we will face a growing number of endometrial carcinomas in aged patients, so the need to deepen all the facets of this specific entity is needed more than ever.

One of the most commonly adopted threshold is set at 65 years old by the World Health Organization (6), although this definition may not be applicable in developing countries (7) and does not encompass additional variables that are crucial while planning the surgical strategy, such as the presence of physiological distress, comorbidity, or polypharmacy summarized in the concept of “ frailty”.

“Frailty” corresponds to a reduction in the psycho-physical reserves necessary to cope with stressors, predisposing the patient to adverse events (8). This is a paramount issue to evaluate considering that surgery is the mainstay of EC treatment at any age.

FIGO (International Federation of Gynecology and Obstetrics) staging guidelines require the execution of total hysterectomy, bilateral salpingo-oophorectomy, and nodal assessment (9) with the minimally invasive approach progressively acquiring a pivotal role in this management (10–13), especially in older patients (14).

Hence, we witnessed a decisive conceptual shift from systematic lymphadenectomy toward sentinel lymph node (SLN) mapping that prospectively showed both a high sensitivity and negative predictive value in view of reduced lymphatic complications (15–17).

Nonetheless, the SLN procedure is burdened by various technical pitfalls.

Lack of surgical experience represents a cross-sectional risk factors for failed mapping (18).

Besides, non-endometrioid histology, enlarged or macro-metastatic lymph nodes, and lympho-vascular space invasion (LVSI) were identified as independent risk factors for unsuccessful mapping, probably due to lymphatic congestion from neoplastic emboli (19, 20).

In this context, some authors claimed age as a potential risk factor but its specific role still needs to be proven (19, 20).

The increased mapping failure imposes a compensatory increase in side-specific lymphadenectomy within the SLN algorithm, but this seems in contrast to the common trend to both surgical and adjuvant undertreatment in the elderly (21–23).

In this scenario, we designed a study with the primary endpoint to assess the overall detection rate, the successful bilateral mapping, and the mapping failure rate comparing women under 65 (Group 1) and over 65 years old (Group 2).

Secondary outcomes were the assessment of SLN anatomical distribution and the identification of predictors for mapping failure.

## Materials and Methods

Patients diagnosed with apparently early-stage EC undergoing minimally invasive SLN biopsy with cervical injection of ICG between May 2015 and March 2021 were retrospectively retrieved.

In this multicentric experience, the participating centers were the Department of Woman and Child Health and Public Health, Fondazione Policlinico Universitario A. Gemelli IRCCS, Università Cattolica del Sacro Cuore of Rome, Italy, as coordinating center (545 patients); the Department of Gynecology and Obstetrics, University of Parma, Italy (115 patients); the Department of Gynecologic Oncology, University of Palermo, Italy (113 patients); and the Department of Gynecologic Oncology, Gemelli Molise SpA, Italy (71 patients).

IRB approval was obtained, and all patients signed written informed consent.

Inclusion criteria were preoperative histological diagnosis of EC, radiological assessment of apparently uterine-confined disease, the minimally invasive approach at the time of surgery (laparoscopic or robotic) (24, 25), and the intracervical stromal injection of IGC.

Exclusion criteria were the presence of bulky pelvic or para-aortic lymph nodes or the evidence of extrauterine dissemination at preoperative CT scan, the cervical injection of tracers other than IGC, the application of radiotherapy or chemotherapy in neoadjuvant setting, and diagnosis of a concomitant primary cancer.

All patients underwent a scheduled preoperative workup including pelvic ultrasound, pelvic examination, chest and abdomen CT scan, hysteroscopic biopsy, and a lower abdomen MRI based on the clinician’s decision (26)

A comprehensive surgical staging was conducted, including minimally invasive total hysterectomy, bilateral salpingo-oophorectomy, SLN biopsy, and eventually peritoneal biopsies or omentectomy if the histology was high grade serous, with or without uterine manipulator (27).

A reflex side-specific pelvic lymphadenectomy was pursued in cases of SLN mapping failure, while the excision of enlarged lymph nodes was selectively performed when required, in line with NCCN algorithm (17).

The procedure started with a diagnostic laparoscopy and lysis of peritoneal adhesion taking care not to enter the retroperitoneal space and interrupt the lymphatic channels draining the uterus.

The cervical stroma was then injected with 4 ml of ICG solution (2 ml at the 3 o’clock and 2 ml at 9 o’clock positions), previously prepared dissolving 25 mg of ICG powder in 20 ml of sterile water.

After 15 min from the injection, we accessed the pelvic retroperitoneum and developed the paravescical and pararectal avascular spaces activating the near-infrared modality to avoid the iatrogenic disruption of lymphatic channels and to clearly identify the pelvic SLN.

The SLN was defined as the first juxtauterine dye-mapping lymph node along a visibly identifiable lymphatic pathway (28). Overall detection rate was calculated considering both unilateral and bilateral mapping. The procedure was considered effective when a clear bilateral visualization of SLN was achieved, while it was deemed as unsuccessful in cases of unilateral mapping or bilateral mapping failure.

SLNs were processed by dedicated pathologists with standard ultrastaging (29) or one-step nucleic acid amplification (OSNA) (30, 31).

The positive lymph nodes were classified as isolated tumor cells (ITC), micrometastasis, or macrometastasis.

Demographic, clinical, and surgical features were registered in a shared electronic database, as well as intraoperative and postoperative complications that were described following the Clavien Dindo Classification (32).

We further stratified patients in risk groups for adjuvant therapy according to ESMO-ESGO-ESTRO guidelines (33).

The anatomical localizations of SLN have been divided into “expected” (external iliac and obturator) and “unexpected” (internal iliac, presacral, common iliac, para-caval, and para-aortic) sites based on the specific frequencies reported in the literature (34).

### Statistical Analysis

Differences in clinical, surgical, and histopathologic factors among the two patient groups were examined.

Comparisons between categorical variables have been performed with *χ* ^2^ test or Fisher exact test when required.

Comparisons between continuous variables have been pursued with Student’s *t*-test when data were normal, and with Mann–Whitney *U* test when data were not normal.

Quantitative variables were described using the following measures: minimum, maximum, median, and range.

Qualitative variables were summarized with absolute and percentage frequency tables.

Predictors of SLN mapping failure were assessed through univariate and multivariable analysis.

Multivariable model was constructed considering all features that were found statistically significant (*p* < 0.05) or with a trend toward significance at the univariate analysis (*p* < 0.10) as independent variables and the bilateral mapping (failure/success) as the dependent variable.

All the calculated *p*-values were two-sided, and significance was set at *p* < 0.05. ORs and 95% CIs were reported.

Statistical analysis was performed using the SPSS version 27.0 statistical package.

## Results

Between May 2015 and March 2021, a total of 844 women with apparently early-stage endometrial cancer were enrolled in the study.

Considering the median age of 64 years old, we subdivided the study population into two main groups: women under 65 (Group 1: 449, 53.2%) and over 65 (Group 2: 395, 46.8%).

Clinical, surgical, and histopathological characteristics are described in **Table 1**.

**Table 1:** Clinical, surgical, and histopathological features of the study population.

Variables	Group 1<65 years old *n* = 449 *n* (%)	Group 2≥65 years old *n* = 395 *n* (%)	*p*-value^*^
	**Clinical features**		
**Previous uterine surgery**	172 (38.3)	99 (25.1)	**<0.001**
**Previous abdominal or pelvic surgery**	268 (59.7)	217 (54.9)	0.185
**BMI kg/m^2^ (median, IQR)**	28.1 (24.0–33.7)	29.3 (26.0–34.2)	0.003^**^
	**Surgical features**		
**Surgical approach**			0.723
LPS	312 (69.5)	270 (68.4)	
Robot	137 (30.5)	125 (31.6)	
**Lymphadenectomy**	148 (33.0)	140 (35.4)	0.448
Pelvic	27 (6.0)	23 (5.8)	0.907
Pelvic and lumbo-aortic	35 (7.8)	33 (8.4)	0.766
**Omentectomy**	55 (12.2)	58 (14.7)	0.300
	**Histopathological features**		
**Histotype**			**<0.001**
Endometrioid	406 (90.4)	314 (79.5)	
Non-endometrioid	43 (9.6)	81 (20.5)	
**Grading**			<**0.001**
1–2	363 (80.8)	273 (69.1)	
3	86 (19.2)	122 (30.9)	
**LVSI**			**<0.001**
No	340 (75.7)	255 (64.6)	
Yes	109 (24.3)	140 (35.4)	
**Tumor diameter**			0.085
<20 mm	158 (35.2)	117 (29.6)	
≥20 mm	291 (64.8)	278 (70.4)	
**Myometrial invasion**			**<0.001**
<50%	326 (72.6)	215 (54.4)	
≥50%	123 (27.4)	180 (45.6)	
**Cervical invasion**			0.050
No	404 (90.0)	338 (85.6)	
Yes	45 (10.0)	57 (14.4)	
**FIGO stage**			**<0.001**
IA	288 (64.1)	171 (43.3)	
IB	62 (13.8)	112 (28.4)	
II	33 (7.3)	38 (9.6)	
IIIA–IIIB	9 (2.0)	11 (2.8)	
IIIC	56 (12.5)	57 (14.4)	
IVB	1 (0.2)	6 (1.5)	
**Prognostic risk groups**			**<0.001**
Low	226 (50.3)	118 (29.9)	
Intermediate	36 (8.0)	61 (15.4)	
High-intermediate	84 (18.7)	88 (22.3)	
High	102 (22.8)	122 (30.9)	
Advanced metastatic	1 (0.2)	6 (1.5)	

^*^ Pearson χ^2^ test.

^**^ Mann–Whitney U test.

BMI, body mass index, LPS, laparoscopy, IQR, interquartile range, LVSI, lympho-vascular space invasion, FIGO, International Federation of Gynecology and Obstetrics.

Statistically significant values have been highlighted in bold.

Concerning clinical features, the two groups mainly differed in terms of “previous uterine surgery” (38.3% Group 1 *vs*. 25.1 Group 2, *p* < 0.001) and BMI (28.1 Group 1 *vs*. 29.3 Group 2, *p* = 0.003), while no differences were noted in the surgical procedures performed.

The surgical staging procedures were always pursued through a minimally invasive technique with 582 (69%) patients treated by laparoscopic and 262 (31%) by robotic approach.

Analyzing the distribution of the histopathological features, patients in Group 2 showed considerably more aggressive biology compared to those in Group 1; indeed, the rates of non-endometrioid tumors (9.6% Group 1 *vs*. 20.5% Group 2), G3 tumors (19.2% Group 1 *vs*. 30.9% Group 2), LVSI (24.3% Group 1 *vs*. 35.4% Group 2), and myometrial invasion ≥ 50% (27.4% Group 1 *vs*. 45.6% Group 2) were significantly higher in patients over 65 than younger counterparts (all *p* < 0.001).

This resulted in a significantly different distribution of the FIGO stage (*p* < 0.001) with more advanced tumors clustered in Group 2.

Similarly, we found an unbalanced proportion of the prognostic risk group among patients <65 and ≥65 years old (*p* < 0.001); i.e., Low-risk tumors were more frequently represented in Group 1 (50.3% Group 1 *vs*. 29.9% Group 2), while Intermediate (8.0% Group 1 *vs*. 15.4% Group 2), High-intermediate (18.7% Group 1 *vs*. 22.3% Group 2), High risk (22.8% Group 1 *vs*. 30.9% Group 2), and Advanced metastatic (0.2% Group 1 *vs*. 1.5% Group 2) were pooled in Group 2.

In **Table 2**, we reported the data on the SLN detection and on the pathological lymph node status both of the entire population and of the population divided into the two age groups.

**Table 2:** SLNs mapping and histopathological findings.

Variables	All *n* = 844 *n* (%)	Group 1<65 years old *n* = 449 *n* (%)	Group 2≥65 years old *n* = 395 *n* (%)	*p*-value^*^
**Overall detection rate (mono-bilateral)**	767 (90.9)	421 (93.8)	346 (87.6)	**0.002**
**Successful bilateral mapping**	610 (72.3)	346 (77.1)	264 (66.8)	**0.001**
**Mapping failure (missing/mono-lateral)**	234 (27.7)	103 (22.9)	131 (33.2)	**0.001**
**Patients with lymph node metastasis**	138 (16.4)	69 (15.4)	69 (17.5)	0.410
Pelvic lymph-node	137 (99.3)	69 (100)	68 (98.6)	
Lumbo-aortic +/- pelvic lymph-node	7 (5.1)	3 (4.3)	4 (5.8)	
No. of patients not staged^**^	17 (2.0)	4 (0.9)	13 (3.3)	**0.012**
**SLN histological status^#^**				0.394
**Negative**	650 (84.7)	361 (85.7)	289 (83.5)	
**Positive**	117 (15.3)	60 (14.3)	57 (16.5)	
ITC	28 (23.9)	15 (25.0)	13 (22.8)	
Micrometastasis	50 (42.7)	31 (51.7)	19 (33.3)	
Macrometastasis	39 (33.4)	14 (23.3)	25 (43.9)	

^*^ Pearson χ^2^ test.

^**^ Patients with no SLN detection and who did not undergo pelvic lymphadenectomy for comorbidity and/or surgeon’s decision.

^#^The analysis was performed on patients in whom at least one SLN was identified (all cases: n = 767; <65 years old n = 421; ≥65 years old n = 346).

SLN, sentinel lymph node; ITC, isolated tumor cell.

Statistically significant values have been highlighted in bold.

The overall detection rate, the successful bilateral mapping and the mapping failure rate of the SLN were respectively of 90.9%, 72.3%, and 27.7% in the whole population.

Analyzing the two groups separately, we showed an increase in the mapping failure rate up to 33.2% in patients over 65 (22.9% Group 1 *vs*. 33.2% Group 2, *p* = 0.001), and simultaneously the successful bilateral mapping and the overall detection rate were significantly decreased (respectively: 93.8% Group 1 *vs*. 87.6% Group 2, *p* = 0.002 and 77.1% Group 1 *vs*. 66.8% Group 2, *p* = 0.001).

No differences in the number of patients with metastatic lymph nodes (pelvic and pelvic and/or lumbo-aortic) were noted in the two groups (respectively: 15.4% Group 1 *vs*. 17.5% Group 2, *p* = 0. 410 and 0.7% Group 1 *vs*. 1.0% Group 2, *p* = 0.711), and even when restricting the analysis to the SLNs, the distribution of positive and negative SLNs was comparable among groups (positive SLNs: 14.3% Group 1 *vs*. 16.5% Group 2, *p* = 0.394).

Specifically, in the node-positive population, we found 28 ITC (23.9%), 50 micrometastasis (42.7%), and 39 macrometastasis (33.4%).

Anyhow, at the time of the pathological staging, we did not consider ITCs as positive lymph nodes due to their still uncertain prognostic value.

Of note, a significantly higher rate of patients in Group 2 were surgically under-staged, due to the lack of application of the SLN algorithm for comorbidity and/or surgeon’s decision (0.9% Group 1 *vs*. 3.3% Group 2, *p* = 0.012).

**Figure 1:**
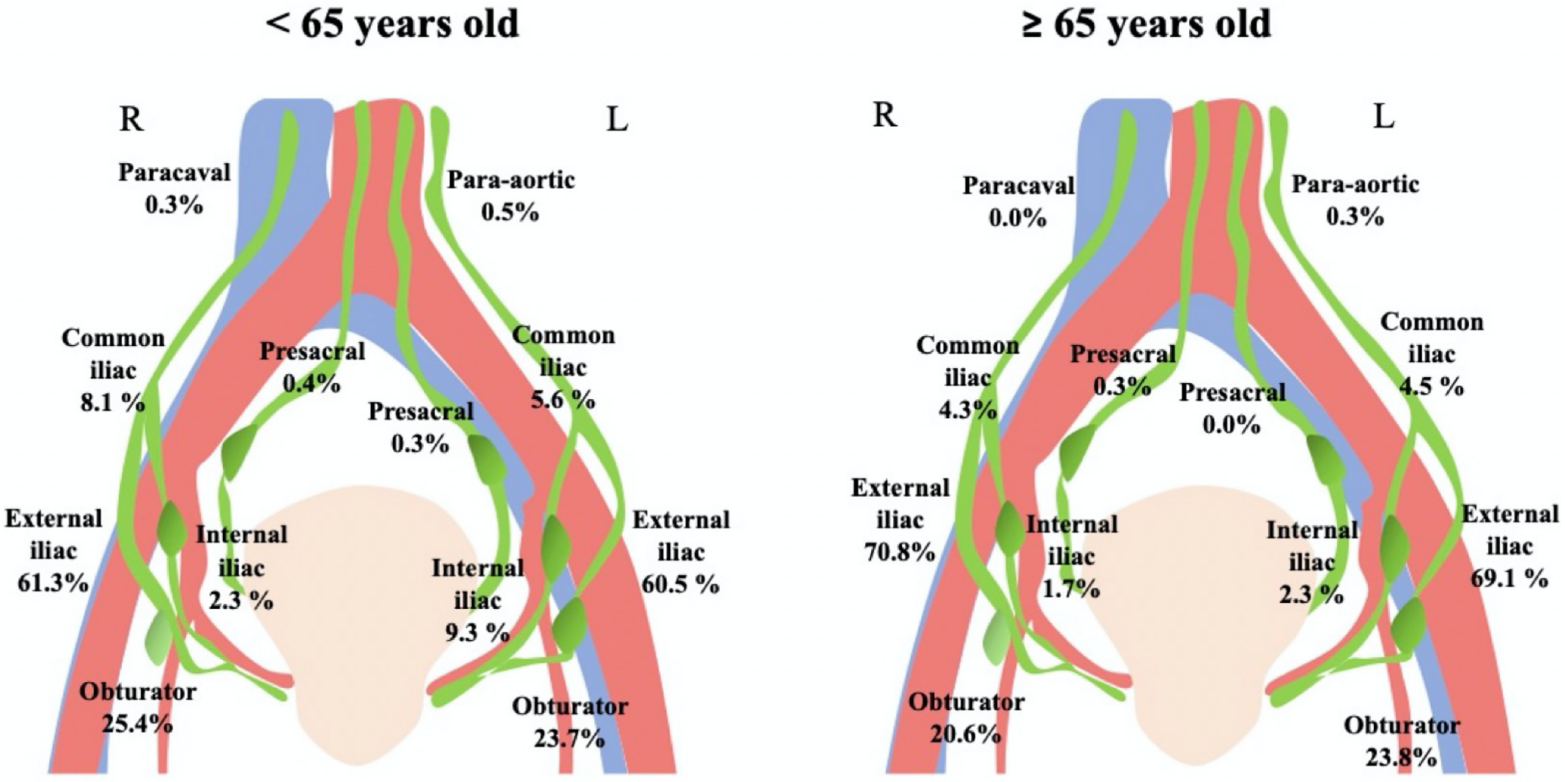
Anatomical localizations of sentinel lymph nodes.

We then analyzed in detail the anatomical locations of the SLNs (**Table 3**, **Figure 1**).

**Table 3:** Anatomical localizations of sentinel lymph nodes.

Variables	All *n* (%)	Group 1<65 years old *n* (%)	Group 2≥65 years old *n* (%)	*p*-value^*^
**Right hemipelvis**	694	393	301	0.058
**Expected site**	616 (88.8)	341 (86.8)	275 (91.4)	
External iliac	454 (65.4)	241 (61.3)	213 (70.8)	
Obturator	162 (23.3)	100 (25.4)	62 (20.6)	
**Unexpected site**	78 (11.2)	52 (13.2)	26 (8.6)	
Internal iliac	28 (4.0)	16 (2.3)	12 (1.7)	
Presacral	4 (0.6)	3 (0.4)	1 (0.3)	
Common iliac	45 (6.5)	32 (8.1)	13 (4.3)	
Para-caval	1 (0.1)	1 (0.3)	0 (0.0)	
**Left hemipelvis**	686	375	311	**<0.001**
**Expected site**	605 (88.2)	316 (84.3)	289 (92.9)	
External iliac	442 (64.5)	227 (60.5)	215 (69.1)	
Obturator	163 (23.8)	89 (23.7)	74 (23.8)	
**Unexpected site**	81 (11.8)	59 (15.7)	22 (7.1)	
Internal iliac	42 (6.1)	35 (9.3)	7 (2.3)	
Presacral	1 (0.1)	1 (0.3)	0 (0.0)	
Common iliac	35 (5.1)	21 (5.6)	14 (4.5)	
Para-aortic	3 (0.4)	2 (0.5)	1 (0.3)	

^*^Pearson χ^2^ test.

SLN, sentinel lymph node.

Statistically significant values have been highlighted in bold.

The “expected” sites (external iliac and obturator) were confirmed to be the most frequent SLN localizations in both Group 1 (Right hemipelvis 86.8%, Left hemipelvis 84.3%) and Group 2 (Right hemipelvis 91.4%, Left hemipelvis 92.9%)

Remarkably, in older women, there was a decreased SLN mapping along the so-called “unexpected” localizations (internal iliac, presacral, common iliac, para-caval, and para-aortic).

Nonetheless, a statistically significant difference between “expected” and “unexpected” SLN localizations was recorded only in the left hemipelvis (unexpected sites: 15.7% Group 1 *vs*. 7, 1% Group 2, *p* < 0.001), while on the right side, only a trend toward significance was detected (unexpected sites: 13.2% Group 1 *vs*. 8.6% Group 2, *p* = 0.058).

Several factors that could hinder sentinel mapping have been evaluated through a univariate and multivariate analysis (**Table 4**).

**Table 4:** Uni- and multivariate analysis for SLN mapping failure.

Variables	Univariate analysis		Multivariate analysis	
	OR (95% CI)	*p*-value	OR (95% CI)	*p*-value
**Age, years**		**0.001**		**0.011**
<65	Reference		Reference	
≥65			1.495 (1.095-2.042)	
**Previous uterine surgery**		0.259		
None	Reference		–	–
Yes	1.202 (0.874–1.653)			
**BMI kg/m^2^**	1.021 (1.000–1.044)	0.054	1.023 (1.000–1.046)	**0.047**
**Histotype**		**0.003**		**0.024**
Endometrioid	Reference		Reference	
Non-endometrioid	1.813 (1.218–2.700)		1.619 (1.067–2.458)	
**Grading**		0.191	–	**-**
1–2	Reference			
3	1.256 (0.892–1.768)			
**LVSI**		**0.014**		**0.044**
No	Reference		Reference	
Yes	1.492 (1.084–2.053)		1.407 (1.010–1.961)	
**Tumor diameter**		0.773	–	**-**
<20 mm	Reference			
≥20 mm	0.954 (0.692–1.314)			
**Myometrial invasion**		0.748	–	**-**
<50%	Reference			
≥50%	0.950 (0.693–1.301)			
**Cervical invasion**		0.114	–	**-**
No	Reference			
Yes	1.425 (0.918–2.213)			

BMI, body mass index; LVSI, lympho-vascular space invasion.

Statistically significant values have been highlighted in bold.

Among the considered variables: age, non-endometrioid histotype, and LVSI appeared to be significantly correlated with mapping failure at univariate analysis, while BMI showed a strong trend towards significance (age ≥ 65 OR: 1.667, 95% CI: 1.230–2259, *p* = 0.001; BMI per 1 unit-increase OR: 1.021, 95% CI: 1.000-1.044, *p* = 0.054; non-endometrioid histotype OR: 1.813, 95% CI: 1.218–2.700, *p* = 0.003; presence of LVSI OR: 1.492, 95% CI: 1.084-2.053, *p* = 0.014).

At multivariate analysis, these factors were further confirmed to be independent predictors of unsuccessful mapping (age ≥ 65 OR: 1.495, 95% CI: 1.095–2.042, *p* = 0.011; BMI per 1 unit-increase OR: 1.023, 95% CI: 1.000–1.046, *p* = 0.047; non-endometrioid histotype OR: 1.619, 95% CI: 1.067–2.458, *p* = 0.024; presence of LVSI OR: 1.407, 95% CI: 1.010–1.961, *p* = 0.044).

**Figure 2:**
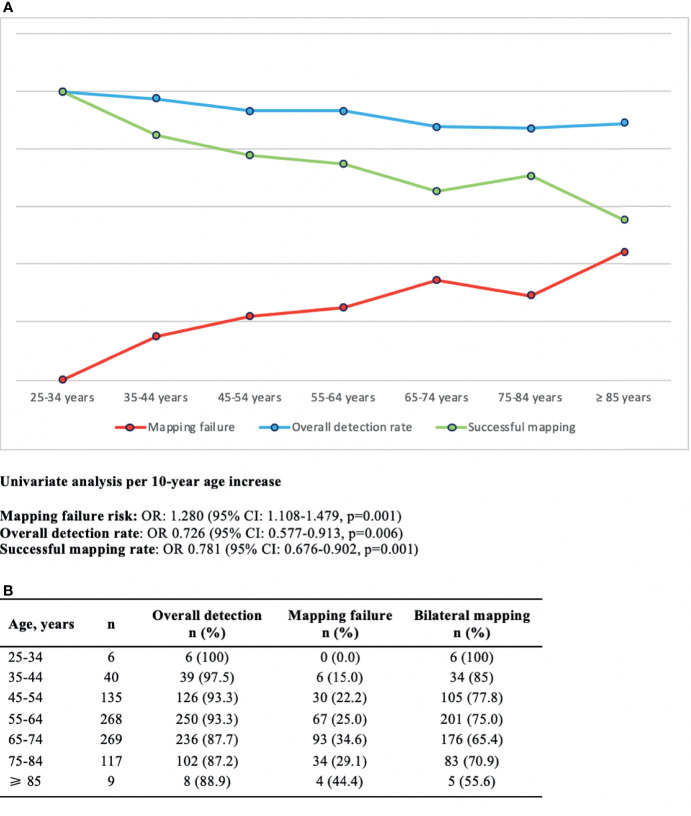
**(A)** Graphical representation of SLN detection rates for 10-year age increase and corresponding binomial logistic regression analysis. **(B)** Frequency table.

Applying binomial logistic regression analysis (**Figure 2**), we found a 1.280-fold increase in the risk of failed mapping for every 10-year-old increase in age (OR: 1.280, 95% CI: 1.108–1.479, *p* = 0.001) which was specular to the decrease of both the overall detection rate and the bilateral mapping (OR: 0.726, 95% CI: 0.577–0.913, *p* = 0.006 and OR 0.781, 95% CI: 0.676–0.902, *p* = 0.001, respectively, for the overall detection rate and the bilateral mapping).

**Table 5** displays intra- and postoperative characteristics of the study population.

**Table 5:** Intraoperative complications, postoperative complications, and adjuvant treatment of the enrolled population.

Variables	All *n* = 844 *n* (%)	Group 1<65 years old *n* = 449 *n* (%)	Group 2≥65 years old *n* = 395 *n* (%)	*p*-value^*^
**EBL, ml (median, range)**	50 (0–1500)	50 (0–1000)	50 (0–1500)	**<0.001^‡^**
**Intra-operative complications**	19 (2.3)	9 (2.0)	10 (2.5)	0.606
Visceral lesions	13 (68.4)	8 (88.9)	5 (50.0)	
Vascular lesions	6 (31.6)	1 (11.1)	5 (50.0)	
**Post-operative complications^#^**				
All grade	41 (4.9)	28 (6.2)	13 (3.3)	**0.047**
I	6 (14.6)	5 (17.9)	1 (7.7)	
II	27 (65.9)	16 (57.1)	11 (84.6)	
IId	2 (4.9)	2 (7.1)	0 (0.0)	
IIIb	6 (14.6)	5 (17.9)	1 (7.7)	
Adjuvant treatment per prognostic risk group^**^				
**Low**	344	226	118	–
FUP	332 (100)	219 (100)	113 (100)	
NA	12	7	5	
**Intermediate**	97	36	61	0.501
FUP	14 (15.1)	4 (11.8)	10 (16.9)	
EBRT/BRT	79 (84.9)	30 (88.2)	49 (83.1)	
NA	4	2	2	
**High-intermediate**	172	84	88	
FUP	14 (8.1)	4 (4.8)	10 (11.4)	0.121
EBRT/BRT	126 (73.3)	64 (76.2)	62 (70.5)	
CHT+RT	29 (16.9)	16 (19.0)	13 (14.8)	
CHT	3 (1.7)	0 (0.0)	3 (1.7)	
NA	0	0	0	
**High**	224	102	122	**0.018**
FUP	12 (5.6)	2 (2.1)	10 (8.2)	
EBRT/BRT	19 (8.8)	5 (5.3)	14 (11.5)	
CHT+RT	164 (75.9)	81 (86.2)	83 (68.0)	
CHT	21 (9.7)	6 (6.4)	15 (12.3)	
NA	8	8	0	
**Advanced metastatic**	7	1	6	0.999^†^
FUP	1 (14.3)	0 (0.0)	1 (16.7)	
CHT	6 (85.7)	1 (100)	5 (83.3)	
NA		0	0	

^*^Pearson χ^2^ test.

^‡^Mann–Whitney U test.

Post-operative complications have been classified using the Extended Clavien–Dindo classification for post-operative complications.

^**^Analysis was performed per single prognostic risk group.

†Fisher exact test.

EBL, estimated blood loss.

Statistically significant values have been highlighted in bold.

Estimated blood loss (EBL) was significantly higher in older women (median 50m l, range 0–1000 ml Group 1 *vs*. median 50 ml, range 0–1500 ml Group 2, *p* ≤ 0.001).

By the way, even if the overall rate of intraoperative complications was statistically overlapping across the two groups (2.0% Group 1 *vs*. 2.5% Group 2, *p* = 0.606), vascular lesions were far more represented in Group 2 (50%) than Group 1 (11.1%).

Surprisingly, postoperative complications were significantly higher in patients <65 years old (*p* = 0.047).

Specifically, according to Clavien-Dindo classification, we reported in the younger group a higher proportion of grade I (17.9% Group 1 *vs*. 7.7% Group 2), grade IId (7.1% Group 1 *vs*. 0% Group 2), and grade IIIb (17.9% Group 1 *vs*. 7.7% Group 2), while grade II complications were proportionally more frequent in Group 2 (57.1% Group 1 *vs*. 84.6% Group 2).

In particular, “severe” post-operative complications consisted in four cases of post coital vaginal-cuff dehiscence and one jejunal perforation in Group 1 and one case of strangulated umbilical hernia requiring an ileal resection in Group 2.

Furthermore, we assigned each patient to a prognostic risk group and reported adjuvant treatment performed to accurately assess any differences in treatment performed based on age.

Compared to patients in Group 1, women over 65 showed a trend toward undertreatment across all risk categories, even if only in high-risk tumors did the difference reach statistical significance (*p* = 0.018).

In particular, 4.8% in Group 1 *versus* 11.4% in Group 2 and 2.1% in Group 1 *versus* 8.2% in Group 2 did not receive any adjuvant treatment respectively in the high-intermediate and high-risk group (*p* = 0.121 and *p* = 0.018).

To note, only one patient (16.7%) with advanced/metastatic disease did not receive chemotherapy due to comorbidities.

## Discussion

Our study shows how the successful bilateral mapping significantly reduces per 10-year increase in age with a mapping failure threshold graphically located above 65 years old (**Figure 2**).

However, we registered overall (90.9%), bilateral (72.3%), and unsuccessful (27.7%) SLN detection rates that are superimposable to the available literature (15, 30).

As a matter of fact, the bilateral dye uptake progressively decreased from 77.1% in patients aged less than 65 to the 66.8% of the older group (*p* < 0.001).

To strengthen this concept, we developed a multivariable model confirming that age ≥65 years old, together with non-endometrioid histology and LVSI, represent an independent predictor of unsuccessful mapping.

Moreover, the advanced age affects the anatomical distribution of the SLN leading to a stepwise reduction of “unexpected” mapping sites.

Furthermore, in this real-life experience, the proportion of surgical under-staging was significantly higher in the older group, although intraoperative and postoperative complications were statistically overlapping.

Even in terms of adjuvant therapy, elderly patients show increased rates of undertreatment stratified by prognostic risk class, especially in high and high-intermediate risk, and this was variably due to comorbidities, clinician’s decision or patient’s will.

As already noticed by and Sozzi et al. (35) (age ≥ 65 OR: 1.8, 95% CI: 1.14–2.98, *p* = 0.012) and Tortorella et al. (20) (OR: 1.41 per 10-year increase in age, 95% CI: 1.08–1.84; *p* = 0.01), older age was linked to unsuccessful mapping but only at univariate analysis.

Probably due to our larger sample size, we were able to design a multivariable model, where controlling for BMI, non-endometrioid histotype, and LVSI, the age ≥65 confirmed to be an independent risk factor for mapping failure (age ≥ 65 OR: 1.495, 95% CI: 1.095-2.042, *p* = 0.011).

The rationale sustaining this finding could be that aged collecting lymphatic channels are enlarged, characterized by the deterioration of their intrinsic contractile pump, and more permeable (36).

Furthermore, tissue fibrosis, pro-inflammatory status, and increased stiffness of the extracellular matrix lead to a reduced production of lymphangiogenic factors and subsequent lymphatic capillary rarefaction (37, 38).

For these reasons, during aging, the lymphatic draining function progressively declines, leading to a decreased uptake of the indocyanine green (IGC) during SLN mapping.

In addition, according to the present literature, elderly women with EC are more frequently diagnosed at an advanced stage, with histologically aggressive tumors (38) and worse immunohistochemical profile marked by a higher expression of mutated p53 protein and decreased E-Cadherin expression (39).

We confirmed this worst clinicopathologic framework, and specifically we found a greater proportion of non-endometrioid histology and LVSI in patients ≥65 years old.

These two histopathological features, meanwhile represent independent predictors for mapping failure, as already emphasized by Sozzi et al. (35).

Therefore, the advanced age is burdened by a double bonding to unsuccessful mapping both directly as an independent factor and indirectly due to a higher incidence of biologically aggressive tumors.

We also pointed out a greater median BMI in aged patients (28.1 Group 1 *vs*. 29.3 Group 2, *p* = 0.003), although this is in contrast to the inverse relationship between age and BMI reported by Lachance et al. (<45 years, 46–64 years, and >65 years with a BMI 40.3, 35.3, and 31, respectively; *p* < 0.001) (40).

If confirmed, this trend toward greater BMI in elderly EC patients should represent an additional independent risk factor for mapping failure.

From an anatomo-surgical point of view, we confirmed that the obturator and the external iliac were the most frequent area of SLN detection in both age groups (34), while the “unexpected” sites reduce proportionally with increasing age.

Furthermore, considering the anatomical model proposed by Persson et al., the external iliac and obturator SLNs are located along the upper paracervical pathway (UPP), while internal iliac and presacral SLNs are located along the lower paracervical pathway (LPP) (41).

We can therefore hypothesize that the accessory infra-ureteral and neural lymphatic pathways (42) constituting the LPP, which more frequently drain to the “unexpected” sites, are also the first to atrophy with advancing age, although this possibility requires prospective validation.

When considered from the perspective of surgical complexity, the ratio of pelvic and lumbo-aortic lymphadenectomies during minimally invasive surgical staging is comparable among the two groups, and also the overall rate of intraoperative complications was overlapping, with no conversion to laparotomy required (43).

However, we should notice that the relative proportion of vascular lesions is greater in the elderly (11.1% Group 1 *vs*. 50.0% Group 2) and consensually the EBL is statistically higher in this subset of patients (*p* < 0.001).

Indeed, as already reported by other studies (44), atherosclerosis and tissue fragility increased the risk of intraoperative vascular injury in aged EC patients (45).

This technical concern is reflected in a greater rate of under-staging that varies from 3.3% in Group 2 to 0.9% in Group 1.

As a matter of fact, the surgeon’s attitude in case of mapping failure in the elderly is more conservative, with a higher tendency not to follow the SLN algorithm, and to omit the side-specific lymphadenectomy (46, 47).

However, as reported by Giannice et al. (48), the inclusion of lymphadenectomy in the surgical management of elderly patients does not significantly affect surgical morbidity.

In addition, several studies demonstrated that both chemotherapy (49) and radiotherapy (50, 51) were feasible and with an acceptable toxicity profile in elderly EC patients.

Despite these evidences, as already widely reported in literature (46), we found a statistically higher rate of adjuvant undertreatment in the older Group, especially referring to high-risk patients.

This implies that elderly patients are less likely to receive the standardized optimal treatment, with a negative impact on their prognosis (22, 23).

Concerning postoperative complication, we surprisingly found a higher rate of major complications in the younger group in contrast to the tendency upon higher complications in the elderly reported by literature (22, 40, 52).

This is possibly due to both the small overall number of events and the occurrence of four post coital vaginal-cuff dehiscence in sexually active younger patients.

Strengths of the study include that, to the best of our knowledge, this is the largest cohort of endometrial cancer patients in which SLN mapping rate was assessed in aged *versus* younger group.

Furthermore, the elderly cohort is often scarcely represented in clinical trials (21, 44, 53).

The main limitation of the study lies in its retrospective design that cannot exclude reporting bias, although the high standardization of preoperative and intraoperative management increases the accuracy of our results.

The reduced rate of bilateral dye uptake with aging imposes the utmost technical attention in order to minimize the procedure-related pitfalls.

In this perspective, the IGC reinjection (41), a gentle retroperitoneal dissection following embryological avascular planes, and focusing on “expected” area of SLN mapping, along the UPP, represent useful practical tricks in this older subgroup of patients.

Reducing the need for pelvic lymphadenectomy, especially in elderly patients, is crucial to lower the potential intra- and postoperative morbidity related to lymphatic complications and senile vascular fragility.

In this scenario, further prospective studies need to be designed with the aim to investigate the prognostic role of side-specific lymphadenectomy in case of non-mapping, especially in this fragile subset of patients.

Nevertheless, age per se does not represent an accurate predictor of morbidity; therefore, the possibility to receive any type of treatments should be gauged on validated onco-geriatric scales, such as the comprehensive geriatric assessment (CGA).

This tool provides a holistic evaluation also of psychosocial and functional proficiency toward the development of a personalized and integrated treatment strategy and long-term follow-up (54).

In light of these considerations, the decision-making algorithm in elderly EC patients should be tailored based on the CGA evaluation and a growing attention should be paid to upgrade the perioperative care programs (Enhanced recovery after surgery/ERAS) aiming to maximize the therapeutic portfolio in aged population (55) and to enhance patient’s quality of life (56).

## Conclusions

Our study is the first to settle an independent correlation between advanced age and reduced uptake of the SLN, with a significant increase in unsuccessful mapping every 10 years.

Old age acts as a risk factor for SLN mapping failure not only intrinsically but also in relation to the greater share of other independent risk factors such as LVSI, non-endometrioid histotype, and BMI.

Furthermore, SLN identification within “unexpected” anatomical areas is less frequent in the elderly, suggesting that the surgeon should target the usual uptake along UPP during the management of the SLN in this subgroup of patients.

These measures can be useful to minimize mapping failure and the consequent risk of surgical under-staging and adjuvant undertreatment.

All the efforts of the scientific community must be directed to guarantee the highest therapeutic standard of care to this fragile subset of patients that will gradually increase in the near future, always with the purpose to push higher up the treatment/morbidity trade-off.

## Data Availability Statement

The raw data supporting the conclusions of this article will be made available by the authors, without undue reservation.

## Ethics Statement

Ethical review and approval required for the study were provided by the Internal Review Boards in accordance with the local legislation and institutional requirements. The patients/participants provided their written informed consent to participate in this study.

## Author Contributions

SC, AR, VV, and FF: writing and data interpretation. VV, AR, and VAC: data analysis. SC, AR, SGA, and AG: study design and literature search. AR, GiuS, VAC, VV, and AG: data collection. All authors: reviewing of the final manuscript. All authors contributed to the article and approved the submitted version.

## Conflict of Interest

The authors declare that the research was conducted in the absence of any commercial or financial relationships that could be construed as a potential conflict of interest.

The reviewer LA declared a past co-authorship with the authors to the handling editor.

## Publisher’s Note

All claims expressed in this article are solely those of the authors and do not necessarily represent those of their affiliated organizations, or those of the publisher, the editors and the reviewers. Any product that may be evaluated in this article, or claim that may be made by its manufacturer, is not guaranteed or endorsed by the publisher.
